# Chinese EFL Students’ Social-Emotional Competence, Grit, and Academic Engagement

**DOI:** 10.3389/fpsyg.2022.914759

**Published:** 2022-06-10

**Authors:** Cheng Zhang, Lizhi Mao, Nanshu Li, Xiaoye Gu

**Affiliations:** ^1^School of Law, Chongqing University, Chongqing, China; ^2^Department of Sports, University of International Business and Economics, Beijing, China

**Keywords:** Chinese EFL students, social-emotional competence, academic engagement, psychology, grit

## Abstract

Regarding the constructive function of students’ academic engagement in learning a foreign language, understanding the individuals’ intrapersonal characteristics effective on engagement has gained attention. To keep up with this line of research, the present study tried to probe the contribution of grit and social-emotional competence to Chinese EFL learners’ academic engagement. To do this, 493 Chinese EFL students, including both males and females, were selected conveniently to participate in the study. For collecting data, a Likert scale questionnaire entailing three items on grit, social-emotional competence, and academic engagement was administered online. Spearman Rho correlation index and multiple regression analysis along with ANOVA were employed to analyze data. The findings revealed a positive and direct relationship between Chines EFL students’ grit, social-emotional competence, and academic engagement. Furthermore, the results showed that compared to social-emotional competence, EFL students’ grit can predict more powerfully academic engagement. The implications of the findings are considered in the present study.

## Introduction

Students’ academic achievement is the main objective of any educational system. Participation and cooperation of all parties is prerequisite for ensuing this academic success and excellence. Besides teachers’ teaching and appropriacy of materials, students’ different abilities and involvement in the learning process are essential (Felder and Henriques, 1995), Astin (1984), in the Students’ Involvement Theory, claims that students’ psychological development will be influenced by their academic involvement. Newman (1992) believes that a student’s engagement includes his/her psychological investment toward understanding, learning knowledge, and mastering skills intended by the education. Like motivation and other prevailing and widespread researched constructs, academic engagement is considered a blooming concept in education (Hiver et al., 2021), in a way that Reschly and Christensen (2012, p. 4) address it as “a new kid on the block.”

Remarkably, recent research on the features of teaching and learning has tried to focus on the pupils’ experiences and their involvement in a particular teaching program rather than evaluating the general teaching and learning (e.g., Durksen et al., 2017). Following this perspective, researchers introduced a conceptual model for academic engagement. It is theorized to encompass four aspects, including “cognitive,” “emotional,” “behavioral,” and “agentic” (Reeve and Tseng, 2011; Reschly and Christensen, 2012; Kahu, 2013). All of the mentioned dimensions relate to students’ Manner of involvement in the tasks, understanding, and performance (Yin, 2018; Zheng and Yu, 2018).

Students’ engagement has been widely researched in several domains and populations (Oga-Baldwin, 2019). Comparing the amount of research addressing the academic engagement of students in math and science and the ways to enhance it (Skinner et al., 2009; Wang and Eccles, 2013), few studies have been done on the EFL field (Yu et al., 2019; Khajavy, 2021), particularly in the Chinese context (Jiang and Zhang, 2021).

Academic engagement of students in the foreign language learning domain is one of the desirable experiences (Khajavy, 2021). Students’ engagement is perceived to be an important requirement in EFL classes (Oga-Baldwin, 2019). Several studies have focused on enhancing academic engagement to enrich education quality and students’ educational success (Christenson et al., 2012). Though, the factors predicting students’ engagement in foreign language learning contexts are under research and need more work (Philp and Duchesne, 2016; Oga-Baldwin, 2019). Taking into account the lack of empirical research on the predictors of academic engagement, the present study tries to tackle this lacuna by bringing into focus the grit and social-emotional competence of Chines EFL students. We hope our study can do its bit to the literature on the effective factors for improving academic engagement in the EFL setting, particularly in the Chinese culture.

There are different occasions in life in which passion and commitment to long-term goals are worthwhile. Given the possibility of facing adversities along the way, having a determination to succeed in defined goals, taking short time or lasting several years are critically important. This determination and persistence to accomplish long-term objectives have been conceptualized as “grit” by Duckworth et al. (2007), which is one of the individual differences. Considering the perseverance and determination for long-lasting objectives as features of grit, it is supposed that grit would reduce disaffection and improve academic engagement. In main focus of the present study is on the predictive role of grit on academic engagement. Previously, Wie et al. (2020), trying to validate an L2 grit scale in the Chinese context, have found a link between Chinese EFL learners’ grit and their socio-biographical variables. In this study, they examined the influence of seven socio-biographical variables (including age, gender, education, and the length of stay abroad, L2 joy, language competence, and multilingualism) on L2 grit, among which Language competence, L2 joy, and age statistically significantly predicted L2 grit.

Besides grit, the other factor assumed to be effective on EFL learners’ academic engagement is their Social-Emotional competence. Social-Emotional Learning (SEL), as an integral constituent of basic education, has recently attracted the researchers’ and educators’ attention (Zhou and Ee, 2012). Through the process of social-emotional learning, a student acquires the required knowledge and skills to act successfully in different social settings. SEL results in Social-Emotional Competence (SEC) which capacitates learners to establish a self-monitor self-regulate ability toward their behavior and their learning, respectively (Wilson et al., 2001). SEC has received outstanding attention in L2 education, and a revival of interest in L2 learners’ emotions, particularly the positive ones is witnessed (Mystkowska-Wiertelak, 2021). With that in mind, researchers, instructors, and curriculum designers are eager to come up with an intervention program to enhance social-emotional competence. Recent studies on the effect of developing SEC on students’ academic life have shown that students with a higher SEC reached higher academic achievements and engaged better in their learning (Durlak et al., 2011; Wang et al., 2019) and were able to communicate positively (Zins et al., 2004; Hall and DiPerna, 2017; Salmela-Aro and Upadyaya, 2020). Regardless of the growing interest in research on the concept of SEC in the educational context, there are a small number of studies on SEC in the EFL context overall and Chinese collectivist culture in particular (Li and Xu, 2019; Chen and Zhang, 2020; Zhou et al., 2021), however, none of them has been done on the relationship between SEC, grit, and EFL students’ academic engagement in the Chinese context.

Considering the paucity of research on the predicting factors of academic engagement in the Chinese EFL context and the malleability nature of grit and SEC, the present study aims to consider the relationship between Chinese EFL students’ grit, social-emotional competence, and their academic engagement by proposing the following questions:

1. Is there any relationship between Chinese EFL students’ social-emotional competence, Grit, and academic engagement?
2. Can Chinese EFL students’ social-emotional competence and grit predict academic engagement?

## Literature Review

### Academic Engagement

Referring to learners’ active involvement in the learning process, engagement works as a learner-initiated path to widely admired educational outcomes like an academic achievement (Christenson et al., 2012). It relates to the range of efforts learners do to move from not understanding and unknowing to understanding and knowing and as a result achievement. Overall, academic engagement represents “what students do to make academic progress” (Reeve, 2013, p. 580).

Christenson et al. (2012) consider a pluridimensional construct with three unconnected but mutually supportive aspects, including behavioral, cognitive, and emotional. In this model, *behavioral engagement* deals with the students’ involvement in the learning process in respect of attentiveness and mindfulness, endeavor, and perseverance; in the EFL context, behavioral engagement is a students’ close attention to information sources, effort, and persistence in confronting setbacks. Oga-Baldwin (2019) believes behavioral engagement is the most fundamental dimension due to its high correlation with academic engagement. *Emotional engagement* points to having positive feelings such as interest and not experiencing negative emotions like anxiety during solving a task (Skinner et al., 2008); considering EFL setting, it is enhancing curiosity and reduces anxiety and irritation. Finally, *cognitive engagement* deals with students’ intentional thoughts and how they employ learning strategies such as elaboration to acquire a piece of knowledge or skill. It, also, refers to EFL students’ sophisticated application of strategies for learning and the run of simulations to mentally recognize problems and solve them (Christenson et al., 2012; Reeve, 2013).

“Agentic engagement” was introduced as the fourth aspect by Reeve and Tseng (2011). Therefore, a four-aspect conceptualization of student engagement was substituted for the three-aspect one. They defined the fourth aspect as “students’ constructive contribution into the flow of the instruction they receive” (p. 258), and considered it “a uniquely proactive and transactional type of engagement” (Reeve, 2013, p. 581). According to Reeve and Tseng (2011), agentic engagement includes examples such as “offer input, express a preference, offer a suggestion or contribution, ask a question, communicate what they are thinking and needing, recommend a goal or objective to be pursued, communicate their level of interest, solicit resources or learning opportunities, seek ways to add personal relevance to the lesson, ask for a say in how problems are to be solved, seek clarification, generate options, communicate likes and dislikes,…” (Reeve and Tseng, 2011, p. 258).

The significance of learner engagement in EFL contexts is more pivotal than in other fields (Mercer and Dörnyei, 2020). To effectively automatize their language skills language, learners need to be involved in the broad training and practice. Furthermore, in task-based and communicative approaches to language teaching, active engagement of learners is highly desirable to boost their participation in interactions and collaborations. L2 engagement is the degree of language learners’ mental or physical involvement in completing a task (Hiver et al., 2021) which is considered a prerequisite for enhancing communicative language ability requiring intensive involvement and practice (Mercer and Dörnyei, 2020). According to language education stakeholders, fostering learners’ engagement is one of the fundamental aspects of promoting EFL learners’ ultimate success (Mercer, 2019). To expand the research on this demand, several studies considered the factors with the potential effect on increasing students’ engagement, for example, rapport and teacher care (Derakhshan et al., 2022), willingness to communicate (Mystkowska-Wiertelak, 2021), enjoyment (Dewaele and Li, 2021), non-verbal credibility behaviors and immediacy (Derakhshan, 2021), employability and stress (Ma and Bennett, 2021), support from parents, teachers, and peers (Chen, 2005), motivation (Yu et al., 2019), and emotions, grit (Khajavy, 2021). However, as Hiver et al. (2020) point out research on EFL learners’ engagement is in its infancy and needs more work. To our knowledge, few studies have on Chinese EFL students’ academic engagement, particularly the potential impact of grit and social-emotional competence on it considering the collectivist culture of China. Hence, it is needed to give thought to their role.

### Grit

First introduced by Angela Duckworth et al. (2007), the personality trait of grit is described as “perseverance and passion for long-term goals” (p. 1087), which is the ability to work vigorously toward difficulties and sustain interest and puissance for a long time even with different adversities and setbacks in their progress. Trying to find an answer to the question “why do some individuals accomplish more than others of equal intelligence?” (Duckworth et al., 2007, p. 1087), they list some non-cognitive attributes of successful individuals including creativity, charisma, emotional intelligence, vigor, and the most common personal quality in every field, i.e., grit. According to Duckworth et al. (2007), a gritty person not only try to complete at hand-works but also make attempt to extend his/her persistence over time. The advantage of these people is their stamina. Regarding it as the most significant factor, Duckworth et al. (2007) believe in Grit as a two-dimensions construct: *consistency of interest* referring to one’s durable passion regardless of disappointments or failures and challenges; and perseverance of effort which points to the person’s inclination toward working strenuously and continuous investment of energy despite confronting challenges.

Besides the crucial contribution of intelligence to enhance academic achievement, Duckworth et al. (2007), argue the vital role of non-cognitive characteristics like persistence. In addition, early literature shows evidence of emphasis on perseverance along with intelligence in accomplishing academic success (Terman and Oden, 1947; Howe, 1999). In spite of rich literature on the positive correlation of grit with constructive psychological outcomes such as academic success and academic persistence (Duckworth and Quinn, 2009; Galla et al., 2014; Strayhorn, 2014), raised levels of academic self-efficacy (Datu et al., 2017), academic adjustment (Bowman et al., 2015), school engagement and motivation (Datu et al., 2018), and self-efficacy (Jordan et al., 2015; Datu et al., 2017), optimal wellbeing (Datu et al., 2016), there are few studies of grit in the EFL setting.

Considering the outstanding contribution of persistence and continued effort to the L2 learning process, the construct of grit has received great attention, particularly in the Chinese context (e.g., Lee, 2020; Wie et al., 2020; Dewaele and Li, 2021). It has been positively associated with L2 performance (Kramer et al., 2018), enjoyment, and willingness to communicate (Teimouri et al., 2020), academic outcomes (Akos and Kretchmar, 2017), motivation, and emotions (Changlek and Palanukulwong, 2015). However, the number of studies on this non-cognitive concept in the EFL setting is wanting, and additional research is required to elaborate our knowledge regarding the effect of grit in the language learning process and EFL learners’ academic engagement.

One outstanding shortcoming is that most of the conducted studies were in general education and in Western societies with individualistic cultures. Henrich et al. (2010) advise the cautious practice of generalizing the findings of Western studies to non-Western contexts since the operational manner of psychological constructs differ according to the culture of the related context (King and McInerney, 2014). It is crucial to replicate these researches to realize where their results are applicable to Eastern countries including China. Therefore, its generalizability to the EFL context and with the collectivistic culture of China is questionable.

### Social-Emotional Competence

The Social-Emotional Learning (SEL) concept was suggested by Goleman (1995). There is little agreement on the operational characterization of social-emotional competence, and this inconsistency shows itself in the various terminology used for this competence such as “emotional literacy” (Park et al., 2003), “social and emotional intelligence” (Salovey and Mayer, 1990), and “social and emotional competence” (Elias et al., 1997). Collaborative of Academic and Social and Emotional Learning (CASEL) is one of the comprehensive, bridging between theoretical and practical poles. Considering ESL as integral to education and development, CASEL defines it as the process through which all young people and adults acquire and apply the knowledge, skills, and attitudes to develop healthy identities, manage emotions and achieve personal and collective goals, feel and show empathy for others, establish and maintain supportive relationships, and make responsible and caring decisions (CASEL, 2022).

It consists of five principal competencies: “the acquisition of skills to recognize and manage emotions, develop care and concern for others, make responsible decisions, establish positive relationships, and handle challenging situations effectively” (Collaborative for Academic, Social and Emotional Learning [CASEL], 2003 cited in Zhou and Ee, 2012, p. 27). According to CASEL (2022), SEL aims to nurture students’ social-emotional competence (SEC), which includes a batch of skills covering both intrapersonal and interpersonal levels. The first level is related to knowing and managing one’s emotions and regulating them. The latter encompasses the abilities to understand others’ emotions and improve care for them, build up positive relationships as well as the skill of making responsible decisions. In other words, this framework introduces five domains: “*self-awareness; self-management; social awareness; relationship management; and responsible decision* making” (DePaoli et al., 2017, p. 11).

With *self-awareness* skills, an individual is competent to notice his/her own strengths and weaknesses, emotions, and the way these may influence one’s performance (Zins and Elias, 2006); Zhou and Ee (2012) argue students’ tend to develop a higher level of emotional self-control and engage in self-regulation if they are raised to know their emotions metacognitively. Therefore, they will be able to make more responsible decisions. *Self-management* pertains to the mastery to control one’s emotions or impetuses. *Social awareness* relates to the aptitude to understand others’ attitudes and perspectives, interpret their thoughts, and appropriately react to their opinions and emotions (Frey et al., 2000). *Relationship management* points to a person’s ability to make relationships and then effectually interact with others (Eckenrode, 2013). Finally, *responsible decision-making* concerns the capacity to conceive ethical, societal, and safety features in decision-making times. Students with such ability can cope rationally and effectively with daily social and academic situations (Collaborative for Academic, Social and Emotional Learning [CASEL], 2003 cited in Zhou and Ee, 2012).

Several studies have confirmed the effect of SEC on the students’ academic life; for example, predicting students’ grades (Elias and Haynes, 2008; Durlak et al., 2011), students’ academic attitudes and emotions (Wang et al., 2016); academic engagement (Zins et al., 2007), interpersonal relationship (Zins et al., 2004; Delay et al., 2016). In China, Yang et al. (2019) studied the impact of SEC on students’ emotions, interpersonal relationships, and academic achievement. They found a predictive power for SEC regarding students’ ultimate accomplishment. Li (2019) examined the relationship between emotional intelligence and positive emotions of English language students in China. The results indicated a correlation between the SEC and the studied variables like learning achievement and foreign language enjoyment. However, there is no study on the predictive power of SEC concerning EFL Chinese EFL students’ academic engagement and its relation to grit. The present study addresses these issues.

## Methodology

### Participants

The participants of the study were 493 EFL students (valid cases) in China who were selected by the convenience sampling method. They were of both genders with different English language proficiency levels with their age ranging from 22 to 30. They were recruited from three universities and colleges in two municipalities in China (Beijing, Chongqing), most of whom majored in English, Korean, translation, and business English in different educational contexts. Before their participation in this study, all the participants sighed their informed consent (Table 1).

**TABLE 1:** Participants’ demographic information.

Demographic information category	*N*	*%*
**Gender**		
Male	229	46.45
Female	264	53.55
Total	493	100
**Level of education**		
Bachelor of arts	431	87.42
Master of arts	52	10.55
Ph.D.	10	2.02
Total	493	100
**Proficiency level**		
Elementary	117	23.73
Intermediate	229	46.45
Upper-intermediate	112	22.71
Advanced	35	7.09
Total	493	100

### Instruments

The data for the study were collected employing a Likert scale, with four separate sections for demographic information, students’ social-emotional competence, Grit, and academic engagement. These scales were adopted from the *Social-Emotional Competence Questionnaire (SECQ)* (Zhou and Ee, 2012), *Grit Scale (GS)* (Duckworth et al., 2007), and *Academic Engagement Scale (AES)* (Reeve, 2013), respectively. The validity of the questionnaires in the Chinese context and their correctness were considered by three experts in the field before conducting the study. The Social-Emotional Competence Questionnaire and Grit Scale consisted of 25 and eight Likert items, respectively. Finally, the questionnaire on students’ academic engagement presented 17 items that measure students’ perspectives on their engagement toward academic affairs with five points (1: Strongly disagree to 5: Strongly agree).

### Data Collection Procedure

To meet the purposes of the study, data were gleaned from three different universities and colleges in Chongqing and Beijing by distributing questionnaires online via Wenjuanxing (an online data-collection program). Altogether, in the middle of October, 493 questionnaires were smoothly and successfully selected. To make the questionnaires clearer and more understandable for 493 EFL learners, the researcher had translated the original questionnaires from English to Chinese and had sent the Chinese version to two scholars to examine possible mistakes. All participants were notified of how to fill out the questionnaire. In addition, informed consent and willingness had been given to participants before they participated in this research. They were free to perform their rights of withdrawal from the questionnaire if they sensed any discomfort at any moment. Then, the collected data were double-checked before they were sent to SPSS for further analysis, which were paving the way for the subsequent probe into the research questions.

## Results

In order to decide on the data analysis, preliminary measurements should be done. The first step is to measure the reliability of the three questionnaires used in this study.

To determine the reliability indices of all three questionnaires, the process of calculation was repeated three times and the outputs of Cronbach’s alpha (Table 2) revealed that the social-emotional competence (SEC) questionnaire (*r* = 0.93), student grit questionnaire (*r* = 0.70), and student academic engagement (SAE) questionnaire (*r* = 0.92) had satisfactory reliability indices.

**TABLE 2:** Questionnaires’ reliability.

Questionnaire	Cronbach’s alpha	*N* of items
SEC	0.93	25
Grit	0.70	8
SAE	0.92	19

One of the ways the researcher used for making a decision about using parametric or non-parametric analysis in a quantitative study is to examine the normality of the data. Table 3 shows the Kolmogorov-Smirnov index which shows that the distribution of data is not normal for any of the variables. The assumption for having a normal set of data is to have a non-significant index of K-S, but the output revealed that the data normality rule is violated for all three measured variables in this study, and a non-parametric analysis should be conducted to calculate the possible relationships among the variables.

**TABLE 3:** Test of normality for SEC, grit, and SAE.

	Kolmogorov-Smirnov^a^			Shapiro-Wilk		
	Statistic	df	Sig.	Statistic	df	Sig.
SEC	0.042	493	0.034	0.978	493	0.000
Grit	0.094	493	0.000	0.962	493	0.000
SAE	0.076	493	0.000	0.976	493	0.000

*^a^ Lilliefors significance correction.*

### The First Research Question

To measure the possible relationship across Chinese EFL students’ social-emotional competence, grit, and their academic engagement, the first research question was posed. Since it was revealed in the previous table that the data are not normal, a non-parametric correlation index (Spearman Rho) was used.

Derived from the correlational rules, the greater the amount of the relationship, the stronger the possibility of a significant relationship. As indicated in Table 4, the relationship between students’ social-emotional competence and their grit is direct and significant (*r* = 0.528, *p* = 0.000). Similarly, the relationship between students’ SEC and their academic engagement is direct and significant (*r* = 0.502, *P* = 0.000). It can be concluded that the higher the amount of students’ SEC, the higher the amount of their grit and academic engagement.

**TABLE 4:** Correlation among SEC, grit, and SAE.

			SEC	Grit	SAE
Spearman’s rho	SEC	Correlation coefficient	1.000	0.528**	0.502**
		Sig. (two-tailed)		0.000	0.000
		*N*	493	493	493
	Grit	Correlation coefficient	0.528**	1.000	0.504**
		Sig. (two-tailed)	0.000		0.000
		*N*	493	493	493
	SAE	Correlation coefficient	0.502**	0.504**	1.000
		Sig. (two-tailed)	0.000	0.000	
		*N*	493	493	493

** Correlation is meaningful at the 0.01 level (two-tailed).

### The Second Research Question

The second RQ concerns the extent to which Chinese EFL students’ social-emotional competence and grit can predict their academic engagement. This measurement was done by running a multiple regression analysis. The following tables were the output of linear multiple regression analysis including, model summary, ANOVA, and coefficient.

As seen in Table 5, based on the number of items and the value of the Likert type, the mean scores, and standard deviation indices were calculated.

**TABLE 5:** Descriptive statistics for SEC, grit, and SAE.

Variables	*M*	SD	*N*
SAE	67.52	9.55	493
SEC	101.24	18.28	493
Grit	29.47	5.71	493

Table 6 provides a model summary for SEC, Grit, and SAE. It was shown that the model, which contains the scores of students’ social-emotional competence and grit, can explain the amount of variance in the dependent variable (students’ academic engagement). This model can explain 36.20% of the variances in the students’ academic engagement.

**TABLE 6:** Model summary for SEC, grit, and SAE.

Model	*R*	*R* square	Adjusted R square	Std. error of the estimate
1	0.601^a^	0.362	0.359	7.65

*^a^ Predictors: (constant), grit, SEC.*

Table 7 examined the assumption that multiple R in the population equals zero (0). The model reached statistical significance (*F* = (2,490) = 138.85, Sig = 0.000, this really means *p* < 0.05).

**TABLE 7:** ANOVA for SEC, grit, and SAE.

Model		Sum of squares	df	Mean square	*F*	Sig.
1	Regression	16264.776	2	8132.388	138.85	0.000^b^
	Residual	28698.206	490	58.568		
	Total	44962.982	492			

*^a^ Dependent variable: SAE. ^b^ Predictors: (constant), grit, SEC.*

To measure whether the independent variables (students’ social-emotional competence and grit) can predict the dependent variable (students’ academic engagement), the sig. column was reported. As shown in Table 8, both of the independent variables are significant predictors. Comparing the predictability power, students’ grit (*B* = 0.349) proved to have a higher index compared to their index of social-emotional competence (*B* = 0.320).

**TABLE 8:** Coefficients for SEC, grit, and SAE.

Model		Unstandardized coefficients		Standardized coefficients	*t*	Sig.
		B	Std. error	Beta		
1	(Constant)	33.382	2.08		15.99	0.000
	SEC	0.167	0.024	0.320	6.99	0.000
	Grit	0.583	0.077	0.349	7.62	0.000

*^a^ Dependent variable: SAE.*

## Discussion and Conclusion

The present study aimed to focus on the correlation among Chines EFL students’ grit, social-emotional competence, and academic involvement. In addition, we attempted to investigate the predictive power of grit and social-emotional competence on students’ engagement in learning tasks and in their academic activities. The results of the study presented a fundamental confirmation of the real existence of a relationship among the three variables of the study. Furthermore, the analysis of the data showed that both grit and SEC can predict the engagement of the EFL students positively and significantly.

The findings demonstrated that grit could positively and directly influence Chinese EFL students’ academic engagement. This finding is consistent with Salmela-Aro and Upadyaya (2020) who found that students with high grit and curiosity get engaged more. The second part of the first research question concerns the relationship between SEC and academic engagement. As with grit, a direct and positive correlation was seen between SEC and Chinese EFL students’ academic engagement. This finding is consistent with the findings of Eriksen and Bru (2022), which may emphasize the usefulness of employing effectual coping strategies in the learning process of a foreign language. Zimmerman (1990) proposes that students with the capacity to exploit social-emotional strategies are more possible to participate actively in their own learning. Fredricks (2011) argues that emotions can act as fuel for academic engagement. As students undergo positive affects like interest and enjoyment, they are apt to show more energy and effort toward learning tasks. Two studies in the Chinese EFL context done by Yu et al. (2015) and Li (2019) indicated the existence of a direct relationship between L2 achievement and social-emotional intelligence. Since engagement in learning is a prerequisite for L2 achievement, it can be inferred that SEC directly influences students’ academic engagement.

The analysis of the data related to the second research question showed that Chinese EFL students’ grit and SEC can predict their academic engagement. Considering the positive and direct correlation between these three variables, finding this predictive value seems rational. Students with a higher level of grit and SEC engage highly in their academic setting and work studiously to reach their academic goals. Encountering failures and hardships, these students have a higher mastery to manage their emotions and make appropriate decisions. Consequently, students with grit and SEC can successfully control their emotions and overcome difficulties in the context as well as the learning process and achieve their objectives. Being interested in understanding which one, grit or SEC, has higher predictive power on academic engagement, it is revealed that although both factors can explain the dependent variable meaningfully, Chinese EFL students’ grit makes a stronger contribution to their academic engagement, however, this difference in predictive value is insignificant and only 0.009.

As usual with every study, the present one suffers from several limitations. The first weakness is related to its research method and data collection procedure. In order to be able to provide an indication of real cause-and-effect or correlation between the variables, longitudinal data are needed. On the other hand, the online collection of data may contaminate the real findings. The next limitation pertains to the generalization of the findings because the data have been collected only from two provinces in China, therefore, the use of the findings should be done cautiously. Moreover, the present study did not investigate the variables and their effects in detail. Future studies can consider how different components of for example SCE correlate with academic engagement or how grit can influence different types of engagement such as emotional engagement.

In spite of the weaknesses, the present study has significant implications. Theoretically, the obtained findings can present concrete examples of the theoretical assumptions related to academic engagement in the Chinese EFL context. In other words, the direct and positive correlation between grit, SEC, and academic engagement empirically indicates the theoretical construct of academic engagement in the Chinese context. Empirically, given the critical role of grit and SEC and their malleability, EFL teachers can make students familiar with and prepare language learners for the possible challenges and hardships of the process of learning a language (Wang et al., 2021). These two features can be improved through instruction and intervention in the educational contexts (Clark and Malecki, 2019).

## Data Availability Statement

The original contributions presented in the study are included in the article/supplementary material, further inquiries can be directed to the corresponding author.

## Ethics Statement

The studies involving human participants were reviewed and approved by the Chongqing University Academic Ethics Committee. The patients/participants provided their written informed consent to participate in this study.

## Author Contributions

All authors have made a direct contribution to this manuscript.

## Conflict of Interest

The authors declare that the research was conducted in the absence of any commercial or financial relationships that could be construed as a potential conflict of interest.

## Publisher’s Note

All claims expressed in this article are solely those of the authors and do not necessarily represent those of their affiliated organizations, or those of the publisher, the editors and the reviewers. Any product that may be evaluated in this article, or claim that may be made by its manufacturer, is not guaranteed or endorsed by the publisher.
